# Phosphodiesterase type 4 anchoring regulates cAMP signaling to Popeye domain-containing proteins

**DOI:** 10.1016/j.yjmcc.2022.01.001

**Published:** 2022-04

**Authors:** Amy J. Tibbo, Delphine Mika, Sara Dobi, Jiayue Ling, Aisling McFall, Gonzalo S. Tejeda, Connor Blair, Ruth MacLeod, Niall MacQuaide, Caglar Gök, William Fuller, Brian O. Smith, Godfrey L. Smith, Grégoire Vandecasteele, Thomas Brand, George S. Baillie

**Affiliations:** a College of Veterinary, Medical and Life Sciences, University of Glasgow, Glasgow G128QQ, UK; b School of Health and Life Sciences, Glasgow Caledonian University, Glasgow, UK; c National Heart and Lung Institute, Imperial College, W12 0NN, London; d Université Paris-Saclay, Inserm, Signaling and Cardiovascular Pathophysiology, UMR-S 1180, 92296 Châtenay-Malabry, France

**Keywords:** POPDC, Popeye Domain containing proteins, PDE, phosphodiesterase, AKAP, A-kinase anchoring protein, PKA, Protein Kinase A, TREK-1, TWIK-related K+ channel 1, NRVMs, neonatal rat ventricular myocytes, UCR1, upstream conserved region 1, ARVMs, adult rabbit ventricular myocytes, SAN, sinoatrial node, APD, action potential duration, CD, contraction duration, POPDC1, PDE4, Cyclic AMP, Action potential, TREK1, Submitted as preprint on bioRxiv https://www.biorxiv.org/content/10.1101/2020.09.10.290825v1.full

## Abstract

Cyclic AMP is a ubiquitous second messenger used to transduce intracellular signals from a variety of Gs-coupled receptors. Compartmentalisation of protein intermediates within the cAMP signaling pathway underpins receptor-specific responses. The cAMP effector proteins protein-kinase A and EPAC are found in complexes that also contain phosphodiesterases whose presence ensures a coordinated cellular response to receptor activation events. Popeye domain containing (POPDC) proteins are the most recent class of cAMP effectors to be identified and have crucial roles in cardiac pacemaking and conduction. We report the first observation that POPDC proteins exist in complexes with members of the PDE4 family in cardiac myocytes. We show that POPDC1 preferentially binds the PDE4A sub-family via a specificity motif in the PDE4 UCR1 region and that PDE4s bind to the Popeye domain of POPDC1 in a region known to be susceptible to a mutation that causes human disease. Using a cell-permeable disruptor peptide that displaces the POPDC1-PDE4 complex we show that PDE4 activity localized to POPDC1 modulates cycle length of spontaneous Ca^2+^ transients firing in intact mouse sinoatrial nodes.

## Highlights

• POPDC1 forms a complex with type 4 phosphodiesterases (PDE4s) in cardiac myocytes.
• POPDC1 binds PDE4 enzymes in the Upstream Conserved Region 1 (UCR1) domain.
• The PDE4 binding motif within the Popeye domain lies in a region that harbours a mutation, which underpins human disease.
• Disruption of the POPDC1-PDE4 complex modulates the cycle length of spontaneous Ca2+ transients in the sinoatrial node.
• Disruption of the POPDC1-PDE4 complex causes a significant prolongation of the action potential repolarization phase.

## Graphical abstract

**Figure:**
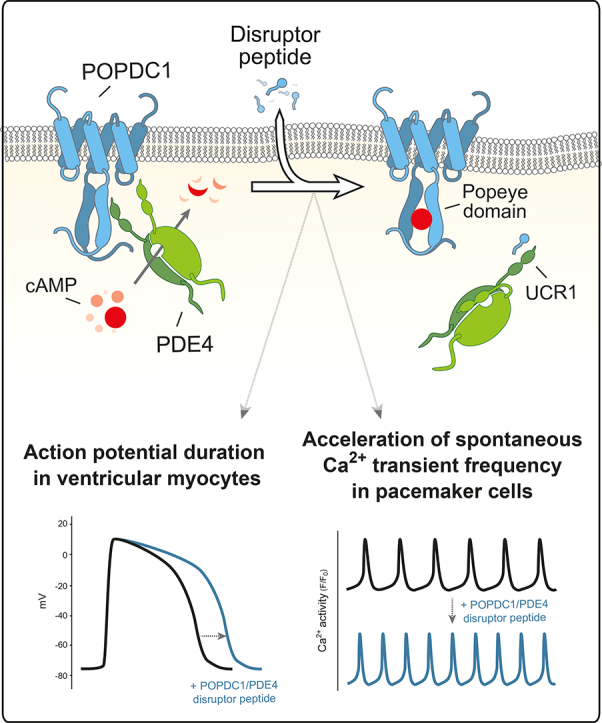

## 1 Introduction

Phosphodiesterases (PDEs) are the only known family of enzymes that can desensitize cAMP signaling via their ability to degrade cAMP. As there are 11 different multi-gene families of PDEs that give rise to over 100 isoforms, which are distributed in a tissue- and cell type specific manner, it has been hypothesized that the precise cellular location of each isoform underpins the function of each PDE species ^1,2^. A good example that illustrates the concept is the PDE type 4 isoform called PDE4D5 [1]. It can be translocated to the vicinity of G_s_-coupled receptors by the signaling scaffold protein beta-arrestin in order to hydrolyze cAMP, while beta-arrestin concomitantly hinders G-protein signaling [2]. This dual functionality is essential for effective desensitization of β-adrenoceptor signaling [3]. PDE4D5 can also anchor to another scaffold protein, RACK1, to localize the enzyme at the leading edge of moving cells to allow direction sensing [4]. Specific disruption of the RACK-PDE4D5 complex hinders cell movement via an exchange factor directly activated by cAMP (EPAC)-mediated signaling mechanism but does not affect β-adrenergic signaling [5]. Conversely, disassembly of the β-arrestin-PDE4D5 complex allows prolonged cAMP signaling following β-adrenergic stimulation that results in hyper-phosphorylation of the β-adrenergic receptor by protein kinase A (PKA) but this action does not affect PDE4D5 located in focal adhesions [6].

The close relationship between PDE anchoring and appropriate activation of cAMP effectors such as EPAC and PKA has been widely investigated in the last decade [7,8]. This is particularly germane in cAMP signaling networks that augment excitation-contraction coupling in the heart during times of increased demand. Highly localized increases in cAMP [9] that appear in cardiac myocytes following β-adrenergic stimulation activate discrete pools of PKA that phosphorylate calcium handling proteins such as the L-type calcium channel, ryanodine receptor (RYR2) and the SERCA regulator phospholamban [10]. Each of these proteins forms a functional complex with an A-kinase anchoring protein (AKAP), which scaffolds PKA and a PDE isoform (reviewed in [8]). This arrangement allows a pulse of cAMP to briefly breach the threshold of activation of the anchored PKA within each complex and deliver a transient augmentation of calcium flux following phosphorylation of the respective PKA substrates. The anchored PDE influences the size and duration of the local PKA drive whilst also guarding against inappropriate activation at resting heart rate [11]. There are many other non-cardiac examples of cAMP effectors (PKA, EPAC and cyclic nucleotide-gated (CNG) channels) that exist in complex with PDEs (reviewed in [8]).

An important, but as yet only partially characterized family of plasma membrane-localized cAMP effector proteins was discovered and named the Popeye domain containing (POPDC) proteins due to their abundant expression in the heart and skeletal muscle [[12], [13], [14]]. POPDC proteins are mainly localized at the sarcolemma and carrying an evolutionary conserved intracellular Popeye domain, which acts as a high-affinity cAMP binding domain [13,15]. Loss-of-function mutations of Popdc1 and Popdc2 in mice and zebrafish established an important role for these genes in cardiac pacemaking and conduction [13,16,17]. Mutations in POPDC1, POPDC2 and POPDC3 have been recently identified in patients suffering from cardiac arrhythmia and/or limb-girdle muscular dystrophy, respectively [[17], [18], [19], [20]]. Moreover, POPDC proteins have also been implicated in atrial fibrillation, long QT syndrome and heart failure [[21], [22], [23]]. Several protein-protein interaction partners of POPDC proteins have been identified including the tandem pore-domain background potassium channel TWIK-related K+ channel 1 (TREK-1). When TREK-1 is co-expressed with POPDC proteins in Xenopus oocytes, the channel displays enhanced membrane trafficking and conductivity, which is sensitive to changes in cAMP levels [13,17,24].

Effector proteins of the cAMP pathway form complexes with PDEs, which limits their activation in response to elevations in cAMP levels ^1^. We show here for the first time that POPDC proteins are similar to other cAMP effectors in that they form a complex with PDE4s. We go on to show that PDE4 activity regulates the ability of POPDC proteins to bind cAMP and to interact with TREK-1 and that disruption of the POPDC1-PDE4 complex significantly reduced the cycle length of spontaneous Ca^2+^ transients in cardiac pacemaker cells under basal conditions. This complex may therefore represent a novel therapeutic target for cardiac arrhythmia.

## 2 Results

### 2.1 PDE4 enzymes and POPDC1 co-localize in ventricular myocytes

**Fig. 1:**
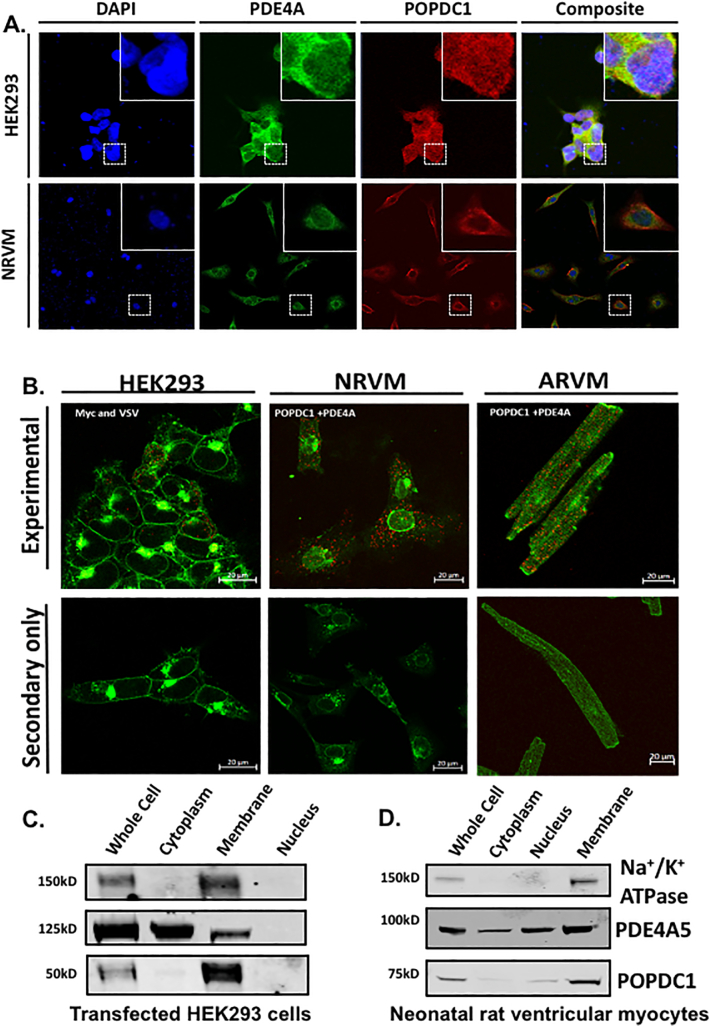
POPDC1 co-localises with PDE4. A. Immunofluorescence showing colocalization of DAPI (nucleus, blue), PDE4A4-VSV (green; polyclonal against human PDE4A) and POPDC1-FLAG (red: polyclonal against POPDC1) in transiently transfected HEK293 (Pearson's coefficient = 0.6., n = 4) and NRVMs (polyclonal against rodent PDE4A, (Pearson's coefficient = 0.6, n = 4).). B. Proximity ligation analysis shows that POPDC1 and PDE4A colocalized in transfected HEK293 cells, NRVMs and ARVMs (upper panels). (same antibodies as in A, n = 4). No background was detected in controls where only the secondary antibodies were used (lower panels). Red dots indicate co-localisation of PODC1 and PDE4A5 and green is wheat germ agglutinin staining to show positions of relevant cells. C, D. POPDC1 and PDE4A5 are detected in the membrane fraction after subcellular fractionation of cell lysate isolated from (C) transfected HEK293 cells or (D) NRVMs (blots typical of experiments done n = 3). (For interpretation of the references to colour in this figure legend, the reader is referred to the web version of this article.)

As PDEs, and in particular PDE4, function as part of “signalosomes” where they restrict the availability of cAMP to cAMP effectors [25], we were interested to see whether the least characterized and newly discovered cAMP effector protein POPDC1 [14] also existed in a complex with PDE4. Initial studies in HEK293 cells that were transiently transfected with POPDC1-FLAG and PDE4A-VSV suggested that the proteins co-localized (Fig. 1A), although overexpression of POPDC1 did cause it to be expressed throughout the cell rather than just in the plasma membrane compartment. This prompted us determine whether this was also the case in neonatal rat ventricular myocytes (NRVMs) where POPDC proteins mainly localize to the plasma membrane [15]. In agreement with previous work, POPDC1 is concentrated in the plasma membrane of NRVMs (Fig. 1A) where it co-localized with a membrane-bound pool of PDE4A. As expected, and previously reported for adult mouse cardiomyocytes [26], POPDC1 was located at the plasma membrane and t-tubules in adult rabbit ventricular myocytes (ARVMs) and showed some co-localisation with PDE4A (Supplementary Fig. 1).The PDE4A5 (murine orthologue of PDE4A4) enzyme is known to have both membrane and cytosolic pools conferred by different targeting cassettes within its sequence [27], in agreement with the observed staining pattern in NRVMs (Fig. 1A) and ARVMs (Supplementary Fig. 1). To verify the specific co-localization of POPDC1 and PDE4A5 we performed in situ proximity ligation assays (PLA), a method we have successfully used in the past to confirm co-localisation of proteins with their binding partners [28],which confirmed the intimate spatial co-segregation of these proteins in HEK293 cells, NRVMs and ARVMs (Fig. 1B, red spots). The co-distribution of PDE4A and POPDC1 in the membrane compartment could also be observed in cellular fractionation experiments using transfected HEK293 cells and NRVMs where, as expected, POPDC1 was found to be mainly present in the membrane fraction, whereas PDE4A was, split between fractions (Fig. 1C and D). A weak POPDC1 signal was seen in case of the nuclear fraction, which is probably due to an expression in the nuclear envelope [29,30]. The integrity of the membrane fraction was validated by using the Na^+^/K^+^-ATPase as a marker (Fig. 1C and D).

### 2.2 POPDC1 interacts directly with PDE4

**Fig. 2:**
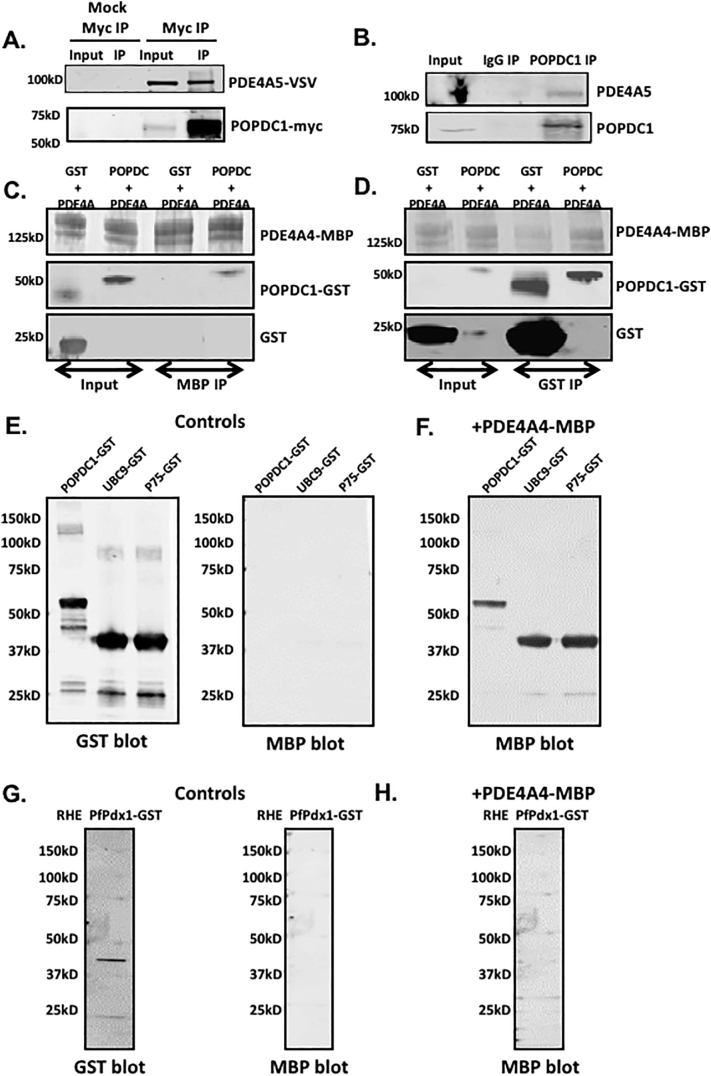
PDE4A and POPDC1 bind directly to each other. A. Myc-immunoprecipitation identifying POPDC1 in a complex with PDE4A5-VSV from transiently transfected HEK293 overexpressing PDE4A5-VSV and POPDC1-myc and B. POPDC1-immunopurification from NRVMs blotted for PDE4A5. C and D Recombinant purified POPDC1-GST and PDE4A4-MBP were mixed at equal concentrations prior to co-immunoprecipitations using amylose resin, that binds to the MBP tag (C.) or glutathione-agarose beads (D.). E. Far Western blotting where recombinant purified POPDC1-GST, UBC9-GST and P75-GST proteins were run on SDS-PAGE and blotted for GST, to ensure the proteins had been successfully transferred, and for MBP, to ensure there was no non-specific binding. F. Membranes were then overlaid with recombinant purified PDE4A4-MBP and re-probed with MBP to detect any interaction between the ‘bait’ proteins and the overlaid PDE4. G and H. A negative control experiment was carried out in the same manner but utilizing recombinant purified RHE PfPdx1-GST protein as it has not been shown to interact with PDE4A4. All blots in Fig. 2 are a representative of experiments done *n* = 3.

To determine whether POPDC1 exists in a complex with PDE4 in a similar fashion to other cAMP-effector proteins, we immunoprecipitated transiently transfected MYC-tagged POPDC1 from HEK293 cells and probed for co-transfected VSV-tagged PDE4A5. We successfully pulled down VSV-PDE4A5 with POPDC1 (Fig. 2A) suggesting a close interaction of the exogenous proteins. Reassuringly, we could also recreate these findings in NRVMs expressing both proteins endogenously (Fig. 2B) suggesting that a pre-formed complex between POPDC1 and PDE4 exists in the cardiac myocytes. Next, we sought to determine whether the interaction between the proteins was direct or mediated by a scaffolding protein. We purified MBP-tagged PDE4A4 (human orthologue of PDE4A5) and GST-POPDC1 and incubated them together. Purifying PDE4A4 using the MBP-tag, also pulled down GST-POPDC1, suggesting that there is a direct interaction (Fig. 2C). Likewise, Isolation of POPDC1 using the GST tag also co-immunoprecipitated MBP-PDE4A4 (Fig. 2D). The assumption that the proteins interacted directly was further verified using far-western techniques, in which immobilized GST-coupled versions of known PDE4A binding proteins UBC9 and P75 NTR [31] and POPDC1 were overlaid with MBP-PDE4A4 (Fig. 2E). All three proteins bound directly to PDE4A4 (Fig. 2F) whereas a control protein RHE PfPdfx did not (Fig. 2G,H), confirming that POPDC1 is able to bind directly to PDE4A4.

### 2.3 Mapping the PDE4-POPDC1 interface using peptide array

**Fig. 3:**
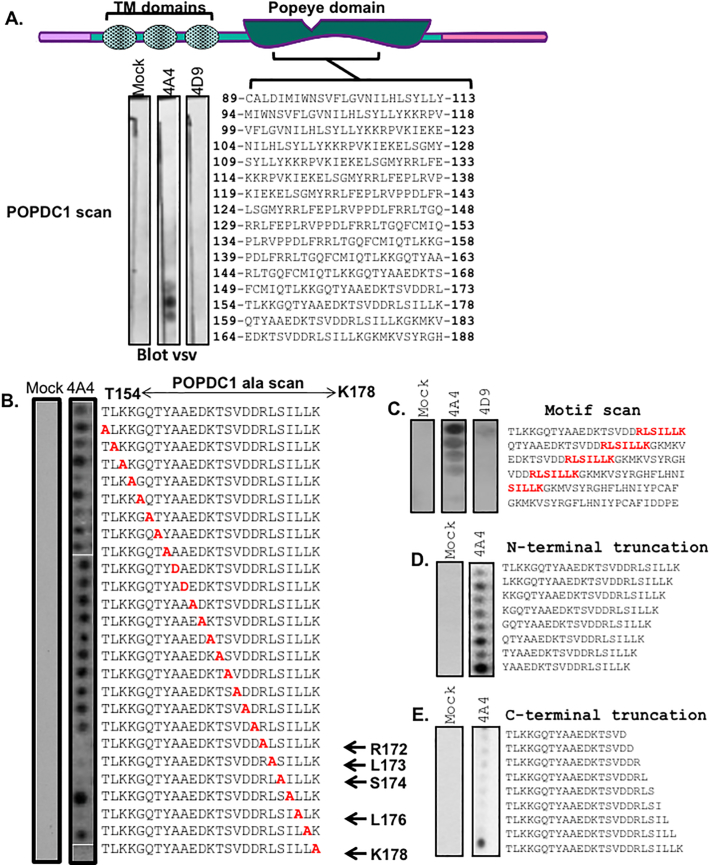
Mapping the PDE4 docking region on POPDC1. A. Peptide arrays encompassing the full sequence of the POPDC1 protein were overlain with lysate from HEK293 cells over-expressing PDE4A4-VSV or PDE4D9-VSV. Each spot contains an immobilized 25mer sequence derived from the POPDC1 sequence. Dark spots indicate peptides that interacted with PDE4 (blotted for VSV tag). B. The initial binding site identified in A. (POPDC1 aa154 – aa178) was further interrogated by alanine scanning to identify single amino acids required for the interaction, conditions as in 3A. C. A motif scan array was constructed to evaluate the importance of the RLSILLK motif discovered in B. This was overlaid with PDE4A4-VSV, conditions as in 3A. D. To confirm whether the binding motif RLSILLK was important for the PDE4 interaction, an N-terminal truncation or E. a C-terminal truncation peptide array was analysed, conditions as in 3A. For all peptide array experiments negative control experiment was performed using mock transfected HEK293 cell lysate.

PDEs are often localized to specific subcellular locations by virtue of protein-protein interactions and peptide array technology has been utilized to define the binding sites that anchor these enzymes [32,33]. We used this technique to map the PDE4A binding site on POPDC1. Initial screening of a library of immobilized peptides of human POPDC1 (25mers shifted by 5 amino acids and encompassing the entire human POPDC1 sequence (UniProt sequence: Q8NE79) suggested a PDE4A4 binding site which is localized within the Popeye domain (Fig. 3A) encompassing amino acids 154 to 178. This site showed very weak to no binding with PDE4D9 (Fig. 3A, right trace and Fig. 3C, right trace). Alanine scanning analysis of this 25mer (Fig. 3B) identified 5 essential amino acids in POPDC1 that are required for interaction with PDE4A4, namely R(172), L(173), S(174), L (176) and K (178). These residues are completely conserved in vertebrates. The positive identification of these residues as points of PDE4A association was verified by a motif scanning experiment where the RLSILLK motif (Fig. 3C, in red) was sequentially shifted through arrays of the POPDC1 sequence of interest. All peptides containing an intact RLSILLK motif showed some degree of binding to PDE4A4 (Fig. 3C). N- and C-terminal truncations of the 154–178 POPDC1 25mer sequence also demonstrated the importance of the RLSILLK motif as binding occurred with peptides that were N-terminally truncated but still retained the RLSILLK domain (Fig. 3D), whereas binding was ablated when the C-terminal residue K(178) was deleted (Fig. 3E).

**Fig. 4:**
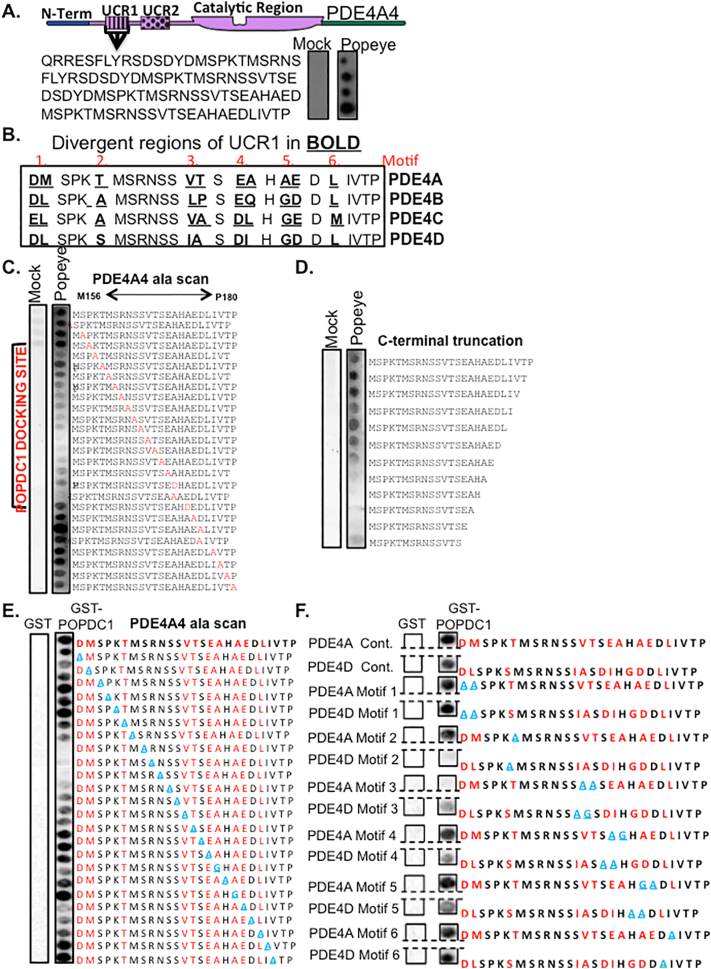
POPDC1 binds to a site within the PDE4 UCR1 domain. A. A peptide array of 25mers shifted by 5 amino acids covering the full PDE4A4 sequence was overlaid with purified recombinant protein consisting of the Popeye domain of POPDC1 tagged with GST. A binding region was identified in the UCR1 domain of PDE4A and the modular structure of PDE4A is shown. A mock control was treated under same conditions but with BSA added instead of GST-Popeye domain. (*n* = 1) B. The Popeye binding domain in UCR1 contains 6 divergent regions (termed motifs 1–6 in red) when sequences from PDE4 A,B,C and are compared. Divergent amino acids are identified in bold and underlined. C. An alanine scanning array of the popeye binding domain was overlaid with purified recombinant protein consisting of the Popeye domain of POPDC1 tagged with GST. Amino acids that are alanised (or changed to Asp if alanine) are shown in red. Control array (Mock) was treated in same manner but the purified Popeye domain was replaced with BSA. D. A C-terminal truncation array was overlaid with purified recombinant protein consisting of the Popeye domain of POPDC1 tagged with GST. Control array (Mock) was treated in same manner but the purified Popeye domain was replaced with BSA. E. An alanine scanning array of the Popeye binding domain was overlaid with purified recombinant protein consisting of full length POPDC1 tagged with GST to identify essential amino acids where dark spots are attenuated. Amino acids that are alanised (or changed to glycine if alanine) are shown in light blue. Divergent amino acids are shown in red. Control array (GST) was treated in same manner but the purified Popeye domain was replaced with GST. Control and test arrays were both blotted for GST F. Spot arrays that compared POPDC1 binding sites from PDE4A and PDE4D overlaid with purified recombinant protein consisting of full length POPDC1 tagged with GST to identify role of divergent sequences (motifs 1–6). Each of the divergent motif was sequentially alanised (or changed to glycine if alanine, shown in light blue) and binding of POPDC1 determined by blotting for GST. Divergent amino acids are shown in red. Control array (GST) was treated in same manner but the purified Popeye domain was replaced with GST. (For interpretation of the references to colour in this figure legend, the reader is referred to the web version of this article.)

PDE4 enzymes are transcribed from 4 genes (A, B, C, and D) that have multiple gene products and approximately 25 unique isoforms exist. PDE4 isoforms have non-redundant functions that are related to differential targeting mechanisms, which place them in specific cellular locations [7]. On overlaying PDE4A4 peptide arrays with purified POPDC1 Popeye domain, we were intrigued to find a POPDC binding domain within the UCR1 region (Fig. 4A). The strongest interaction was detected with a peptide spanning M156 to P180 of PDE4A4 (Fig. 4A, bottom spot), which contains a region whose amino acid sequence is less stringently conserved than most of UCR1 (Fig. 4B). This region has 6 regions of variance which we named “motifs 1–6” (Fig. 4B). A similar experiment overlaying purified full-length POPDC1 revealed a similar binding domain in UCR1 (Fig. 4E). Alanine scanning of the M156 to P180 25mer revealed an essential region that encompassed K159 to H172 of PDE4A4 (Fig. 4C). This region was also shown to be essential for POPDC1 binding in C-terminal truncation analysis (Fig. 4D). Comparison of the cognate regions taken from the two most highly expressed cardiac PDE4s, PDE4A and PDE4D [34] showed that sequences from the PDE4A subfamily gave the most robust interactions with the purified full-length POPDC1 (Fig. 4F, upper two array spots) suggesting that the divergence in the UCR1 sequence in this area underpins POPDC1's preference for PDE4A over other subfamilies. Sequential substitution of each divergent motif on PDE4A and PDE4D UCR1 sequences showed that POPDC1 binding to PDE4D could be attenuated when motifs 2, 3, 4 and 5 were mutated but reductions of POPDC1 binding to PDE4A sequences was only observed when motif 3 was substituted (Fig. 4F).

**Fig. 5:**
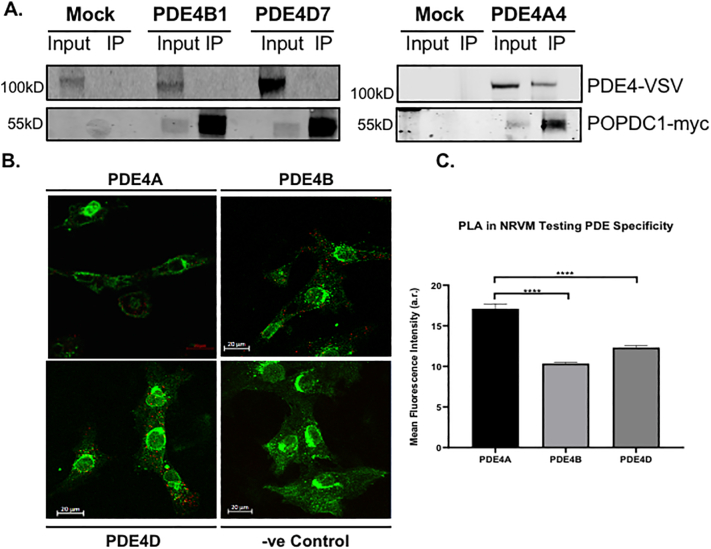
POPDC1 interacts preferentially with the PDE4A sub-family. A. Co-immunoprecipitations were done using HEK293 cell lysate transiently transfected with POPDC1-myc and PDE4A4-VSV, PDE4B1-VSV or PDE4D7-VSV. Myc-agarose resin was used to precipitate POPDC1 and any interacting PDE4 was detected using VSV tag. Western blotting for both myc confirmed pull down of POPDC and VSV confirming successful transfection of PDE4 isoforms. Negative control immunoprecipitation experiments were performed using cell lysate from mock transfected HEK293. B. Specificity of the POPDC1 interaction for endogenous PDE4 isoforms was examined in NRVMs using PanPDE4A, PDE4B and PDE4D antibodies by PLA analysis. Red dots indicate co-localisation. C. Images were quantified using ImageJ. The intensity of the red (PLA) signal was taken for each cell by drawing around each individual cell using the wheat germ agglutinin as the cell boundary. Data represents an n of 3 with each n consisting of 15–20 cells. Significance was evaluated using a one-way ANOVA with Tukeys post-hoc analysis, ****p = 0.0001, compared to PDE4A control. (For interpretation of the references to colour in this figure legend, the reader is referred to the web version of this article.)

Since UCR1 is present in all long PDE4 isoforms, we decided to further investigate the specificity for PDE4A. Data from co-immunoprecipitations performed using transfected HEK293 cells complemented the data from initial peptide array studies (Fig. 3A) that suggested a degree of PDE4A selectivity as neither of the long isoforms PDE4D7 or PDE4B1 was found to interact with POPDC1 using this particular technique (Fig. 5A). As transfected constructs can often mislocalize when over-expressed, the preferential binding of PDE4A binding to POPDC1 was also determined in NRVMs using a proximity ligation assay (PLA). A robust PLA signal (red dots) was detected between POPDC1 and PDE4A (Figs. 1D and 5B). In comparison to PDE4A, significantly weaker signals (red dots per cell) were recorded for POPDC1 interactions with endogenous PDE4B and PDE4D (Fig. 5B and C) reinforcing the notion that PDE4A was the “preferred” partner due to the optimal POPDC1 binding region in the UCR1 divergent region motif 3 (Fig. 4B). A similar relationship was also observed in ARVMs (Supplementary Fig. 2) where PDE4A5 antibodies produced more PLA signals per cell than antibodies against PDE4B. The least were seen for PDE4D. This situation has similarities to the association of PDE4D5 with beta-arrestin-2 where all PDE4s can interact with the scaffold but PDE4D5 is “preferred” due to an optimized binding motif within the PDE's N-terminal region [1].

### 2.4 Functional relevance of the POPDC1-PDE4 complex determined by targeted disruption

**Fig. 6:**
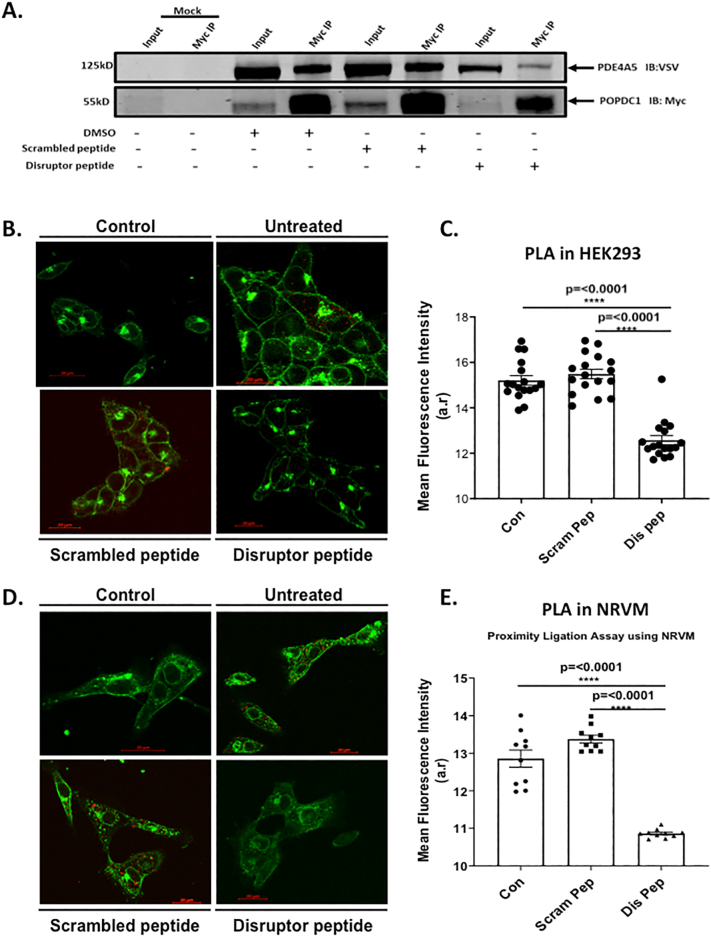
A cell penetrating disruptor peptide dissociates the POPDC1-PDE4 complex. A. HEK293 cells transiently transfected with POPDC1-myc and PDE4A5-VSV were treated with 10 μM scrambled or disruptor peptide. Co-immunoprecipitations of POPDC1 and PDE4A5 performed using Myc-conjugated resin was used to evaluate ability of scrambled or disruptor peptide to compete with PDE4-POPDC1 interaction. The figure is a representative example chosen from three replicate experiments. B. HEK293 cells transiently transfected with POPDC1-myc and PDE4A5-VSV were treated with 10 μM scrambled peptide or disruptor peptide prior to fixation. After PLA staining of HEK293 cells, images were analysed using ImageJ. C. Significance was evaluated using a one-way ANOVA with Tukeys post-hoc analysis, ****p = 0.0001, compared to untreated and scrambled peptide controls. D. The experiment was repeated in NRVM staining endogenous proteins. E. After PLA staining of NRVMs, images were analysed in ImageJ for quantification of PLA signal per condition. The intensity of the red (PLA) signal was taken for each cell by drawing around each individual cell using the wheat germ agglutinin as the cell boundary. Data represents an n of 3 with each n consisting of 15–20 cells. Significance was evaluated using a one-way ANOVA with Tukeys post-hoc analysis, ****p = 0.0001, compared to untreated control and scrambled peptide control. (For interpretation of the references to colour in this figure legend, the reader is referred to the web version of this article.)

Since the localization of individual PDE4 isoforms influences their function, the targeted disruption of PDE4 protein-protein interactions can be a highly specific way to investigate the roles of these enzymes [25]. Each PDE4 isoform may possess many interaction partners and hydrolyze cAMP at a variety of microdomains within a cell. We hypothesized that a cell-penetrating disruptor peptide that could selectively displace the small fraction of the total PDE4 pool that forms a complex with POPDC1 should have a measurable effect on the function of POPDC1. As we have done for other PDE complexes [35,36], we utilized the peptide array information to inform our design of a suitable POPDC1-PDE4 disruptor (Fig. 3) with the sequence TLKKGQTYAAEDKTSVDDRLSILLK (T154-K178 of POPDC1) conjugated to a stearate group to direct trans-membrane transport. A cell-permeable, scrambled peptide was also created using the same amino residues present in the disruptor peptide in a randomized order, and the sequence checked via BLAST to ensure it did not match natural sequences found in other proteins (TTLYTDSSVLKGKRLQDKEKALADI). Co-immunoprecipitations performed with transiently transfected HEK293 cells treated with the disruptor peptide showed a marked reduction in the amount of PDE4A interacting with POPDC1 when compared with those treated with the scrambled control (Fig. 6A). This data suggests that the disruptor peptide was successful in competing against the endogenous PDE4-POPDC1 interaction. This was verified in both transiently transfected HEK293 cells (Fig. 6B, C) and NRVMs (Fig. 6D, E) where disruptor peptide treatment (but not scrambled control peptide) significantly reduced the association of PDE4A and POPDC1 detected by PLA.

**Fig. 7:**
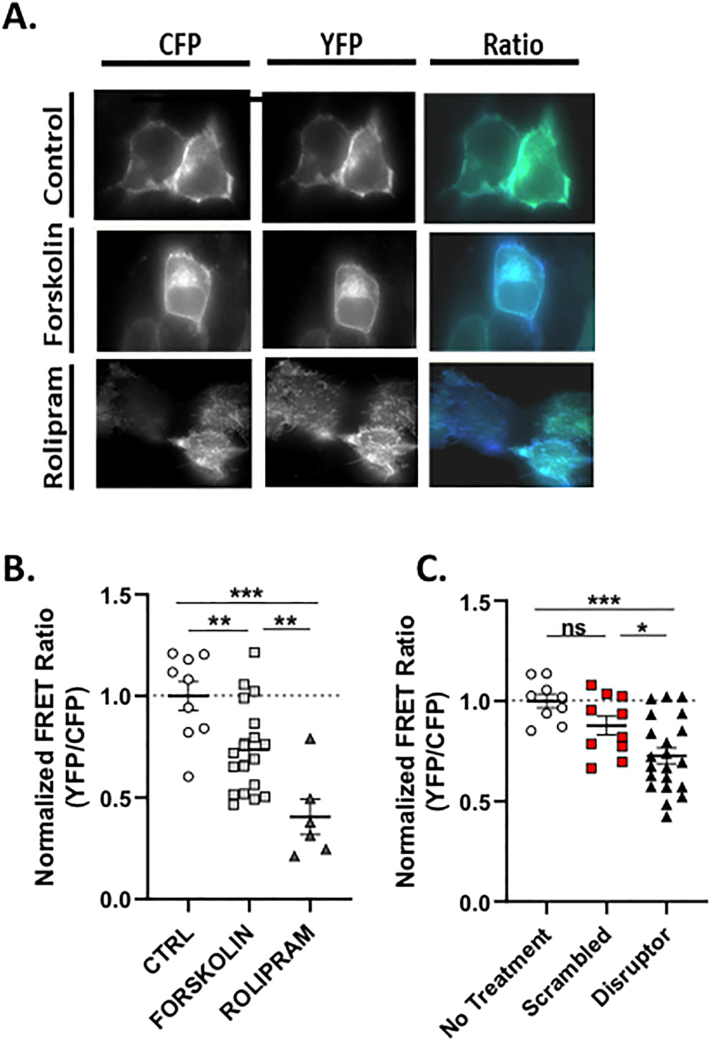
Disruption of the POPDC1-PDE4 complex reduces TREK1 binding. A. HEK293 cells stably expressing PDE4A4 were transiently transfected with POPDC-CFP and TREK1-YFP. After treatment with 25 μM Forskolin or 10 μM Rolipram, static measurements were taken every 5 s for 300 s per cell. Test data was normalised to mean data from an identical but untreated control group of cells. B. FRET ratio calulated as mean ± SEM, control *n* = 9 cells, forskolin *n* = 15 cells, rolipram n = 15 cells per experiment (*n* = 3). Significance was evaluated using a one-way ANOVA with Tukeys post-hoc analysis, ** *p* ≤0.005, ***p = 0.001, compared to control C. Calculation of FRET ratio after treatment with 10 μM scrambled peptide or 10 μM disruptor peptide. Static measurements were taken every 5 s for 300 s. Background fluorescence was subtracted from mean intensities and are plotted as mean subtracted intensities. Results represented as mean ± SEM, untreated *n* = 11 cells, scrambled peptide n = 11 cells, disruptor peptide *n* = 20 cells per experiment (n = 3). A one-way ANOVA with Tukey's post-hoc was used to analyze the FRET ratios.

As POPDC1 is a cAMP binding protein that binds to and regulates the activity of TREK channels [17], and PDE activity can be modulated by protein and lipid binding partners [7], we set out to determine whether POPDC proteins could regulate the activity of PDE4 enzymes. As expected, the activity of purified PDE4A4 was susceptible to the PDE4 inhibitor rolipram (Fig. Suppl. 4B) but the activity did not change when incubated with different concentrations of purified POPDC1 protein (Fig. Suppl. 4A). POPDC1 did not affect the activity of PDE4A nor did it significantly change the rolipram dose response curve (Fig. Suppl. 4B) suggesting that POPDC1's association with PDE4 did not occlude the enzyme's active site. In addition, POPDC1 did not affect the ability of PKA (Fig. Suppl. 4C) to phosphorylate PDE4A4 as measured with either an antibody to PKA phospho-substrates (Fig. Suppl. 4D,E & F) or against Phospho-UCR1(Fig. Suppl. 4D,E & G). Phosphorylation by PKA activates PDE4 long forms, a mechanism that is partially responsible for the transient nature of cAMP responses [37]. The fact that POPDC1's interaction with PDE4A does not seem to alter its ability to hydrolyze cAMP suggests that the POPDC1-PDE4A complex is constitutively formed to protect POPDC1 from inappropriate activation under basal conditions. To test this hypothesis we used an established bi-molecular FRET sensor consisting of POPDC1-CFP and YFP-TREK1 [13] to evaluate the influence of PDE4 activity on the POPDC1-TREK interaction. As expected, increases in global cAMP induced by Forskolin decreased the interaction between POPDC1 and TREK (Fig. 7 A, B). Interestingly, inhibition of PDE4 using the PDE4 specific inhibitor rolipram significantly decreased POPDC1-TREK binding to a greater extent than Forskolin (Fig. 7A, B), suggesting that the PDE4 “pool” that is sequestered to POPDC1 has a highly influential role in “gating” the access of cAMP to PODC1 in order to modulate the POPDC1-TREK interaction.

Next, using the cell-permeable disruptor peptide described above (Fig. 6), we demonstrated that selective disruption of the POPDC1-PDE4 complex could significantly decrease the POPDC1-TREK interaction (Fig. 7 C), presumably due to an increase in cAMP binding to POPDC1 resulting from a higher local cAMP concentration in the vicinity of POPDC1 due to the displacement of PDE4. Treatment with the scrambled peptide control did not produce any significant changes in the POPDC1-TREK interaction as measured by the bi-molecular FRET reporter construct (Fig. 7 C). We were able to detect endogenous expression of POPDC1 and TREK in ARVMS (Supplementary Fig. 3).

**Fig. 8:**
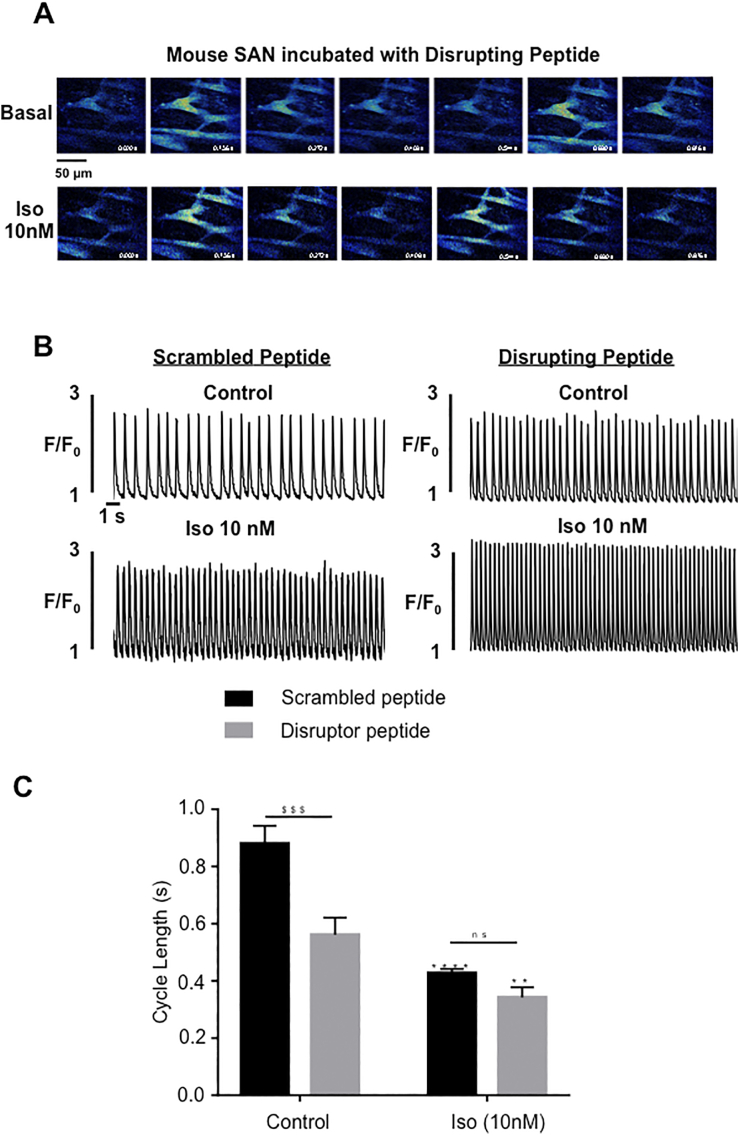
POPDC1-PDE4 disrupting peptide accelerates spontaneous Ca^2+^ transient frequency in isolated mouse SAN (A) Examples of spontaneous Ca^2+^ fluctuations recorded by 2-D confocal imaging in Fluo-4-loaded individual SAN cells embedded within the intact SAN. The SAN was incubated for 1 h with 5 μM disrupting peptide prior to Ca^2+^ measurements in normal Tyrode solution (Control) and in the presence of isoproterenol (Iso, 10 nM). (B) Representative traces of spontaneous Ca^2+^ transients in SAN cells incubated with 5 μM scrambled peptide (left) or 5 μM disrupting peptide (right) in control and Iso 10 nM conditions. Fluorescence traces are expressed as F/F_0_, where F is the fluorescence signal and F_0_ the diastolic fluorescence. (C) Average cycle length (time between two consecutive spontaneous Ca^2+^ peaks) in 6 SAN incubated with 5 μM scrambled peptide and 6 SAN incubated with 5 μM disrupting peptide in control and Iso 10 nM. Bars are mean values ± SEM. **: *p* < 0.01; ****: *p* < 0.0001; ^$$$^: *p* < 0.001. Two-way ANOVA followed by a Sidak's post-hoc test.

Both POPDC1 and PDE4 are highly expressed in the SAN and *Popdc1* and *Popdc2* KO mice display a stress-induced sinus bradycardia. [13,16,17,38]. Since the POPDC1-PDE4 disruptor peptide disassociates the POPDC1-PDE4 complex (Fig. 6) and interferes with POPDC1-TREK1 interaction (Fig. 7), we investigated whether it could impact SAN function. For this, SAN were isolated from adult mouse hearts, loaded with the Ca^2+^ indicator Fluo4, and incubated with the POPDC1-PDE4 disruptor peptide or the scrambled control. Spontaneous Ca^2+^ transients in SAN pacemaker cells were then recorded using 2-D confocal imaging at baseline and following application of 10 nM of the β-adrenergic agonist isoprenaline (Iso, 10 nM, Fig. 8A). In both groups, Iso induced a significant reduction in the cycle length of spontaneous Ca^2+^ transients as expected (Fig. 8B and C). Interestingly, the basal cycle length was significantly lower in SAN treated with the disruptor peptide when compared with those treated with the scrambled peptide, suggesting that POPDC1-PDE4 interaction modulates SAN activity (Fig. 8B and C).

We also evaluated changes in functional output of ARVMs utilizing the CellOptiq® platform (Clyde Biosciences Ltd.; Glasgow, UK). The action potential duration of ARVMs was determined following treatment with the POPDC1-PDE4 disruptor and scrambled control peptides. Measurements of the action potential duration during the action potential repolarization phase (APD 30, 50, 75, 90) were collected from cells which were paced at 1 Hz under baseline conditions (Supplementary Fig. 5A, representative trace shown in Supplementary Fig. 5B). No changes were observed at any time point during repolarization for the scrambled peptide in comparison to the untreated control group (Supplementary Fig. 5 C—F). However, there was a small but significant prolongation of the repolarization phase at APD_30_, APD_50_, APD_75_ and APD_90_ in the presence of the disruptor peptide (one-way ANOVA, post-hoc: Tukey's multiple comparison *p* < 0.0001) (Supplementary Fig. 5 C,D,E,F). The prolongation of the APD at 30, 50, 70 and 90% suggests that disruption of the POPDC1-PDE4 interaction causes small alterations in repolarizing currents.

Frequently, changes in action potential duration are met with changes in contractility [39]. As such, we sought to determine whether the changes in action potential duration correlated with any alterations in cardiomyocyte contractility. Using the parameter contraction duration 50 (CD_50_), the time between 50% upstroke and 50% downstroke is determined, meaning that both contraction and relaxation are considered. Similar to the APD measurements, there was no alteration between the CD_50_ of myocytes treated with the scrambled peptide cells in comparison to the untreated control cells (Supplementary Fig. 6A, B). However, treatment with the disruptor peptide also resulted in no significant changes in CD_50_ time compared to the two control conditions (representative traces Supplementary Fig. 6A, quantification in Supplementary Fig. 6B). So, although action potentials are elongated by the disruptor peptide, this did not manifest itself as a change in myocyte contractility.

## 3 Discussion

PDE4 activity regulates many crucial cAMP-driven processes in a variety of different tissue types even though their expression level is often low. This apparent contradiction can be explained by the discrete cellular localization of individual PDE4 isoforms, which are sequestered to specific micro-domains in cells by virtue of “postcode” motifs that confer protein-lipid interactions with membranes or protein-protein interactions with a variety of scaffolds. PDE4 containing complexes can be spatially defined or have the ability to translocate to areas of high cAMP levels [25]. Once in the correct subcellular domain, PDE4s shape cAMP gradients to allow receptor-specific signals to be relayed via cAMP effector proteins that often exist in close proximity to the phosphodiesterase [8]. Aberrant cAMP signaling caused by mutations in cAMP signaling intermediates, PDE4s, their scaffolds or the associated cAMP effector can result in pathologies such as acrodysostosis, ischemic stroke or McCune-Albright syndrome [[40], [41], [42]].

Here we report for the first time that the most recently discovered cAMP effector, POPDC1, can also be found in a complex with PDE4. Interestingly, the binding site for PDE4 on POPDC1 (R172-K178) contains the residue arginine 172 which is mutated to histidine in a family presenting with dilated cardiomyopathy (DCM) [43]. The conclusions from studies investigating the mechanistic effects of this mutation were that cAMP binding affinity of POPDC1 was not significantly affected, nor were there changes in the subcellular localization of the protein or in POPDC1's ability to interact with TREK1 [43]. Our data suggests the phenotype of the POPDC1^R172H^ mutation may instead be a result of the inability of POPDC1 to bind to PDE4 and thus protect itself from inappropriate cAMP binding under basal conditions. Indeed, in experiments where we tried to phenocopy this situation by using a cell permeable peptide that dislocated PDE4 from POPDC1, we were able to record decreased interaction between POPDC1 and TREK1 (Fig. 7), a prolongation of action potential duration (Supplementary Fig. 5) and an increased frequency of spontaneous Ca^2+^ transients in pacemaker cells from SAN explants (Fig. 8). Our data provides a putative mechanism that could contribute to the disease pathology of the POPDC1^R172H^ mutation. Although it is currently not known whether POPDC2 and POPDC3 also bind PDE4, mutation of leucine 155 to histidine in POPDC3, which corresponds to leucine 173 in POPDC1, was recently identified in a patient suffering from limb-girdle muscular dystrophy (LGMDR26, OMIM 605824). [20] Thus, in at least two of the POPDC proteins, mutations in the putative PDE4 interaction motif are associated with heart or skeletal muscle phenotypes.

Another noteworthy conclusion from our research is that POPDC1 can bind several PDE4 enzymes but has a preference for PDE4As. As the POPDC1 binding site has been mapped to the conserved UCR1 region of PDE4, it is likely that only the UCR1 containing PDE4 long forms associate with POPDC1. The activity of PDE4 in the human myocardium can be attributed equally to PDE4A and PDE4D isoforms [44]^,^ [34] and preference for PDE4A binding by POPDC1 must stem from either the ability of both proteins (POPDC1 and PDE4A) to bind membranes, which may bring them into close proximity (Fig. 1) or a preferential binding motif within the UCR1 domain of PDE4A compared to other sub-families of PDE4 (PDE4B,C and D (Fig. 4). Fine mapping of the POPDC binding site in PDE4 UCR1 identifies a region S162 to S169 (human PDE4A4 numbering) that is completely conserved in all longform PDE4s apart from residues V167 and T168, which are PDE4A specific. This core region must underpin the association of POPDC1 with longform PDE4s. The VT di-peptide cassette is only one of the 6 small, divergent, one or two amino acid stretches that are found in that part of UCR1 (Fig. 4B). Preliminary data comparing POPDC1 binding to PDE4A vs PDE4D sequences where each highlighted divergent region is sequentially substituted with alanine (or Ala to Gly substituted if the amino acid of interest is alanine) shows that PDE4A binds POPDC1 better than PDE4D and is less reliant on the divergent sequences than PDE4D. In fact, when considering each of the six highlighted divergencies between PDE4A and PDE4D, only mutation of the VT dipeptide (and none of the other 5) completely ablates PDE4A binding. This suggests that the preference for PDE4A may lie in this motif. A similar situation exists for the arrestin-PDE4 complex where all PDE4s can associate with the scaffold protein at a site within its N-domain but preference for PDE4D5 is attributed to an additional, specific docking domain in the arrestin C-domain [45]. Whether PDE4 binding by POPDC1 is shared by the other members of the POPDC family remains an open question. The core sequence identified in POPDC1 is also present in POPDC2 and POPDC3 with some conservative substitutions. It will be interesting to test whether the POPDC2 and POPDC3 also preferentially bind PDE4A, or whether they differ in their binding preference.

The observation that POPDC1 and PDE4 co-localize in heart tissue leaves the possibility that this signaling complex also underpins cAMP transduction events in other tissues open. POPDC1 is expressed most abundantly in striated muscle, i.e. heart and skeletal muscle, and these tissues are affected in patients harboring POPDC1 mutations [[17], [18], [19]]. However, POPDC1 is also found in smooth muscle cells, the gastrointestinal tract, uterus and bladder, neurons of the central and autonomous nervous system and epithelial cells [12,46,47]. As PDE4A is also ubiquitously expressed, it is likely that the POPDC1-PDE4 interaction may also be important in other organs and cell types.

*Popdc1* and *Popdc2* are highly expressed in the SAN and loss of function mutations in mice are causing a stress-induced sinus bradycardia phenotype ^13^. We therefore tested whether disrupting the PDE4-POPDC1 interaction might have an effect on cardiac pacemaking. Interestingly, we observed an acceleration of the Ca^2+^ transient frequency after the addition of the disruptor peptide to SAN explants in mice. These data indicate that the POPDC1-PDE4 interaction has a functional relevance in pacemaker cells. The difference in cycle length of Ca^2+^ transients of SAN explants treated with disruptor versus control peptide disappeared after isoproterenol stimulation, suggesting that the POPDC1-PDE4 interaction is more relevant under basal cAMP levels. However, since basal cAMP is much higher in SAN cells compared to atrial or ventricular cells [38] our results do not exclude a role of POPDC1-PDE4 interaction during β-AR stimulation in the working myocardium. PDE4A, B and D isoforms are found in both the rabbit and mouse SAN [38,48]. Currently, the role of individual PDE4 isoforms in SAN function is not fully defined. However, the analysis of cardiac pacemaking in PDE4 null mutants in mice suggest that PDE4B controls intrinsic SAN automaticity and PDE4D might be important for autonomic nervous system-mediated regulation of heart rate and conduction. However, no specific function in cardiac pacemaking has been assigned for PDE4A, the isoform displaying the strongest interaction with POPDC1.

The fact that the PDE4-POPDC1 disruptor peptide was able to prolong the repolarization phase of the action potential in ARVM but did not affect contraction duration is surprising as they are usually coupled. However, in agreement with this observation, *Popdc1* null mutant mice display deficiencies in cardiac pacemaking but no effect on cardiac contractility has been reported [13]. A well-characterized interaction partner of POPDC1 is TREK-1. In co-injected *Xenopus* oocytes, POPDC1 can enhance TREK-1 current, which is thought to be mainly mediated by increased membrane trafficking of the potassium channel [13], although effects on channel gating have also been observed [17]. The prolongation of the action potential duration after disruptor peptide treatment could at least in part be explained by the modulatory role of POPDC1 on TREK-1 [49]. Binding of TREK-1 to POPDC1 has been mapped to the proximal part of the Popeye domain (residues 132–186) [17] and thus, is either in proximity or overlap with the PDE4 binding site (residues 172–179). TREK-1 current is potently inhibited by intracellular increases in cAMP via PKA [50,51]. Two PKA consensus motifs are present in the C-terminus of TREK1, which also binds AKAP79/150 [52]. Thus, POPDC1 forming a complex with TREK-1 and PDE4 may either be adjacent to, or part of the AKAP79/150 -PKA complex. POPDC1 could be involved in fine-tuning the effect of adrenergic receptor stimulation on TREK-1 current. Interestingly, TREK1 has recently been implicated in cardiac pacemaking and a cardiomyocyte-specific loss of TREK1 causes a stress-induced sinus bradycardia in mice [53]. Thus, loss-of-function mutations of both *Popdc1* and TREK1 display overlapping phenotypes of SAN dysfunction. In contrast to the bradycardia observed in vivo, but in line with the acceleration of spontaneous Ca^2+^ transients we observed in SAN cells with the POPDC1-PDE4 disrupting peptide, SAN cells lacking TREK1 display an increased firing rate of spontaneous action potential [53]. It is therefore tempting to speculate that the effects of the disruptor peptide on spontaneous Ca^2+^ transients result from increased membrane excitability based on the POPDC1/ TREK1 interaction. However, SAN automaticity is generated through multiple voltage-gated ion channels and transporters in the plasma membrane of SAN myocytes collectively called the ‘membrane clock’ and functionally linked to a Ca^2+^ clock mechanism, which involves the local Ca^2+^ release from the sarcoplasic reticulum [54]. It is likely that several ion channels and other electrogenic proteins involved in cardiac pacemaking are interacting with POPDC proteins and probably collectively being responsible for the acceleration of the SAN frequency in response to the application of the disruptor peptide. In this regard it is noteworthy, that the sodium calcium exchanger NCX has recently been identified as an interacting protein of POPDC2 and the loss of *NCX1* was shown to cause SAN dysfunction with a phenotype similar to the one observed in *Popdc1* null mutants (55, 56). HCN4 channels are primarily responsible for I_f_, an important pacemaker current, which drives slow diastolic depolarization and contributes to the spontaneous pacemaker cell activity [57]. However, I_f_ was not affected in the *Popdc2* null mutant [55,56]. Numerous other membrane and membrane-associated proteins also interact with POPDC1 including caveolin3 [26], dystrophin [17], dysferlin [17], ZO-1 [58], VAMP3 [59], NDRG4 [60], GEFT [61], XIRP1 [62] and Annexin 5 [62]. However, a functional association of these proteins with APD or cardiac pacemaking has not yet been established. More work will be required to explain how the loss of PDE4-POPDC1 interaction may cause an acceleration of spontaneous Ca^2+^ transients and a prolongation in APD.

POPDC1 in cardiac myocytes is mainly found in different plasma membrane compartments including the lateral sarcolemma, t-tubules, caveolae and the intercalated disk [14]. However, after transfection POPDC1 is mainly found intracellularly in both NRVM and HEK293 cells (Fig. 1). Similar results have previously been obtained after transfection of POPDC1 into Cos7 cells [12]. It is unclear why transfected POPDC1 is mislocalised but this is probably related to some limiting factor required to support export from the endoplasmatic reticulum and to promote plasma membrane trafficking. In this regard it is interesting that impaired membrane trafficking of POPDC1 was also observed in muscle biopsies of patients carrying *POPDC1* mutations ^17–19^. Possibly heteromerization with POPDC2 is required for membrane trafficking and this hypothesis is supported by the fact that impaired trafficking in patients carrying *POPDC1* mutations was also observed for POPDC2 ^17–19^. Further work will be required to elucidate the molecular control mechanism involved in controlling subcellular localization. However, irrespective of the subcellular localization in both cardiac myocytes and HEK293 cells, a robust co-localization pf POPDC1 with PDE4 isoforms was observed. In western blotting experiments (Fig. 1) a weak but significant nuclear localization was observed for POPDC1. In agreement with this observation, previous work reported localization of POPDC1 and POPDC2 at the nuclear envelope and the nucleoplasm [63]. It is possible that at each of these subcellular locations different PDE4 isoforms are co-localized with POPDC proteins, modulating the interaction with target proteins.

In summation, we present data to suggest that a novel signaling complex containing POPDC1 and PDE4 proteins modulates Ca^2+^ transients firing in the SAN under basal conditions. The findings expand the list of functions ascribed to anchored PDE4s and underpin the importance of POPDC proteins in cardiac excitability.

## 4 Materials and methods

### 4.1 HEK-293 cell culture

Human embryonic kidney (HEK) 293 cells were purchased from ATCC and HEK-293 PDE4A4 stable cell line established by Millipore. HEK-293 and HEK-293 PDE4A4 cells were cultured in high-glucose Dulbecco's Modified Eagle's Medium (DMEM) supplemented with 10% (*v*/v) foetal bovine serum, 1% (v/v)penicillin/streptomycin (P/S), 1% (v/v) l-Glutamine, 1% (v/v) minimum essential media non-essential amino acids (NEAA). HEK-293 PDE4A4 cell media is further supplemented with 500 μg/ml G418 (Promega). Culture conditions were 37 °C in a humidified atmosphere with 5% CO_2_. Cells were split at 80–90% confluence and media was replaced every two-three days as required.

### 4.2 Preparation of ventricular myocytes from neonatal rat and adult rabbit

The preparation of neonatal rat cardiomycoytes was modified from the protocol described by in [64]. 1–3-day old neonatal Sprague-Dawley rats were sacrificed by cervical dislocation and femoral artery dissection. Cardiomyocytes were dissociated from the ventricles by serial digestions with 0.3 mg/ml Type-2 Collagenase (Worthington) and 0.6 mg/ml pancreatin (Sigma) in a balanced salt solution (120 mM NaCl, 20 mM HEPES, 5.5 mM glucose, 5.4 mM KCl, 1 mM NaH_2_PO_4_ and 0.8 mM MgSO_4_ (pH 7.4)). Tissue was then incubated for 20 min at 37 °C in a water bath shaking at 200 cycles per minute. This digestion step was repeated 3–5 times or until the tissue was completely digested. Cell suspensions were collected and pelleted by centrifuging for 5 min at 1250 rpm. The pellet was resuspended in 2 ml of New-born Calf Serum (NCS). Cells were kept at 37 °C in a humidified incubator with an atmosphere of 95% air and 5% CO_2_. After final digestion the cell suspensions were pooled and centrifuged for. The pellet was resuspended in M1 plating media (67 ml D-MEM; 25 mM HEPES (Invitrogen), 17.5 ml M-199 (Invitrogen), 10 ml Horse serum, 5 ml new-born calf serum, 1 ml 200 nM glutamine and 0.1 ml penicillin-streptomycin). As non-cardiomyocytes become attached easily, the cells were pre-plated on 10cm^2^ dishes (Corning) for 2 h to allow differential attachment of non-myocardial cells. Non-adhered cells were collected and centrifuged at 1250 rpm for 5 min. Cells were counted and plated at a density of 1 × 10^6^ cells per well of a 6-well plate that was pre-coated with sterile 1% (*w*/*v*) gelatin (Sigma-Aldrich). For immunocytochemistry, cells were seeded at 1.5 × 105 on mouse lamin (BD Bioscience) (100 μg/ml per coverslip). ARVMs were isolated as described in (Donahue et al., 1998).

### 4.3 Transfection, Western blotting and Immunoprecipitation

DNA plasmid constructs used for transfection included myc-POPDC1, VSV-PDE4A5, VSV-PDE4A4, VSV-PDE4D7, VSV-PDE4B1, CFP-POPDC1 and YFP-TREK1. Plasmid DNA was transiently transfected into HEK-293 and HEK-293 PDE4A4 stable cell lines using Lipofectamine LTX Reagent (Thermo Fisher Scientific) using manufacturers protocol. Cells were transiently transfected with myc-POPDC1 and one of the PDE constructs or CFP-POPDC1 and YFP-TREK1. Cellular lysates were prepared in either 3 T3 lysis buffer (50 mM NaCl, 50 mM NaF, 25 mM HEPES, 5 mM EDTA, 30 mM sodium pyrophosphate, 10% (*v*/v) glycerol, 1% (v/v) Triton X-100; pH 7.5) or a CO-IP buffer (50 mM Tris; pH 8.0, 150 mM NaCl, 2 mM EDTA, 1% (v/v) Triton X-100, 0.25% (w/v) bovine gelatin (Sigma-Aldrich)). Both lysis buffers were supplemented with phosphatase and Complete™ EDTA-free protease cocktail inhibitor tablet (Roche). Protein concentrations of the lysates was determined using the Bradford assay and all samples were equalised for protein concentration.

Proteins were separated using SDS-PAGE (4–12% Bis-Tris gels) and then transferred onto nitrocellulose membranes for Western blotting. For immunoprecipitation, POPDC1(BVES), Myc or VSV were used to immunoprecipitate endogenous, and overexpressed POPDC1 or PDE4 isoforms respectively. The resulting immunocomplexes were captured using Protein G sepharose beads (Invitrogen) at 4 °C overnight on a rotator. The immunocomplexes were collected by centrifuging at 1000 x g for 5 min and washed 5 times in CO-IP lysis buffer. The bound protein complexes were eluted in 2x Laemmli buffer and subjected to SDS-PAGE and immunoblotting. Negative controls using isotype-matched IgG from the same species as the antibodies used were performed in order to screen for non-specific binding. Proteins were visualised using appropriated Alexa Fluor conjugated secondary antibodies scanned using the Odyssey Infrared Imaging System (LI-COR Biosciences, UK) for fluorescence detection of the secondary antibodies. Fluorescence signal intensity was quantified using the Odyssey application software (LI-COR Biosciences, UK).

### 4.4 In vitro pull-down assay

Equal molar concentrations of purified recombinant GST-POPDC1 or His-POPDC1 and MBP-PDE4A4 were mixed in 3 T3 lysis buffer [25 mM Hepes, 2.5 mM EDTA, 50 mM NaC1, 50 mM NaF, 30 mM sodium pyrophosphate, 10% (v/v) glycerol, 1% (v/v) Triton X-100, pH 7.5, containing Complete™ EDTA-free protease inhibitor cocktail tablets (Roche)]. Samples were incubated end-on-end with gentle agitation for 1 h at 4 °C. Pre-equilibrated Ni-NTA Superflow resin (Qiagen) or glutathione sepharose resin (Amersham Biosciences) were added to the protein mixture and incubated with gentle agitation for another 1 h at 4 °C. Beads were then sedimented by centrifugation at 10,000 x*g* for 5 min followed by three washes in 3 T3 lysis buffer. Proteins were then resolved by SDS-PAGE and immunoblotted using GST (Sigma Aldrich), 6His (Sigma Aldrich) or MBP (Abcam) antibodies.

### 4.5 Far Western blotting

POPDC1, UBC9, P75NTR and RHE PfPdx1 were expressed as N-terminal GST (glutathione S-transferase) fusion proteins and purified to homogeneity as previously described. 10 μg of each was run on a gel and transferred to nitrocellulose before being overlain with 5 μg of 4A4-MBP (1 μg per ml) for 4 h. Gels were probed with antibodies against GST and MBP as indicated in figure legend.

### 4.6 SPOT synthesis of peptides and overlay experiments

This was carried out as described by us in detail elsewhere [32].

### 4.7 Immunostaining

For immunofluorescent labelling, cardiac myocytes (NRVMs, ARVMs) were plated onto laminin-coated sterile glass coverslips and HEK293 cells were seeded onto laminin-free cover slips. HEK293 cells were transiently transfected with Myc-POPDC1 and VSV tagged PDE4A5, PDE4B1 or PDE4D7 constructs. After 24-h incubation on coverslips the cells were fixed using 4% (*w*/*v*) paraformaldehyde for 1 h at room temperature with gentle agitation. Coverslips were incubated with wheat-germ agglutinin for 10 min at 37 °C to stain the membranes of the cells. Coverslips were washed three times with PBS and blocked for 1 h at room temperature in blocking buffer (PBS supplemented with 0.5% BSA and 0.25% Triton X-100). Primary antibodies; BVES (Santa Cruz, sc-374081), PDE4A (in-house) for cardiomyocytes and Myc (Abcam) and VSV (Sigma Aldrich) for HEK293, were added at a 1:500 or 1:1000 dilution in blocking buffer to the coverslips and incubated overnight at 4 °C in a humidity chamber. After washing three times in PBS, Alexa-Fluor antibodies (donkey anti-Mouse 488, goat anti-rabbit 488 and donkey anti-rabbit 546 (Invitrogen) were added at a 1:200 dilution in blocking buffer. Cells were washed a further three times before being mounted to glass slides using mounting media containing DAPI nuclear stain. Endogenous PDE4 from Sub-families A,B and D were detected using in-house antibodies raised against the conserved C-terminal regions of each sub-familiy as previously published by us ^34^. Cells were visualised using a Zeiss 5 (LSM) 510 Meta Confocal Microscope and analysis done using Zeiss Image Studio to produce intensity line graphs and Pearson's coefficients. Images were acquired with Zeiss LSM Image Examiner and analysed on ImageJ.

### 4.8 Proximity ligation assay

HEK293 cell were transiently transfected with Myc-POPDC1 and one VSV tagged PDE4 isoform; PDE4A5, PDE4B1 or PDE4D7. NRVMs and ARVMs were stained using BVES antibody (sc-374081, Santa Cruz) and an in-house made PanPDE4A, PDE4B or PDE4D antibody. Prior to carrying out the protocol cells were treated with either 10 μM disruptor peptide or 10 μM scrambled peptide for two hours. To visualise protein-protein interactions in both endogenously and overexpressing cells in situ, Duolink® proximity ligation assay (PLA, Sigma-Aldrich) was employed using the manufactures protocol.

### 4.9 Fluorescence resonance energy transfer

CFP-POPDC1 and TREK1-YFP sensors used within this work were produced and trialed by Froese and colleagues [13]. HEK293 cells were plated on sterilised 24 mm coverslips (VWR) at a low density to allow for single cells to be measured and clear background regions were available to record. Transient transfections with FRET sensors were carried out 48 h prior to imaging according to previous protocols [13].

The coverslip was carefully removed from the culturing dish with watchmaker's forceps and inserted into a metal ring and sealed securely. Coverslips were washed three times with FRET saline (125 mM NaCl, 5 mM KCl, 1 mM Na_3_PO_4_, 1 mM MgSO_4_, 20 mM HEPES, 5.5 mM glucose, 1 mM CaCl_2_, pH 7.4). A 300 μL bath of FRET saline was applied preventing the cells from drying out. The cells were then visualised on an Olympus IX71 inverted microscope under 40× or 60× immersion lenses (Zeiss). Image acquisitions were initiated, and real time measurements were taken. Stimulation with the AC activator, 25 μM forskolin (adenylyl cyclase activator) or inhibition by 10 μM Rolipram (PDE4 inhibitor) was carried out by diluting the drugs in 300 μL FRET saline to allow for total dispersion. For peptide experiments, cells were treated with; 10 μM scrambled peptide, 10 μM disruptor peptide or DMSO, 2 h prior to imaging. Static measurements were taken in 5 s intervals for a total of 300 s to reduce the effects of photobleaching. A beam splitter was used to separate CFP and YFP so that images could be used to obtain a ratio of CFP to YFP in defined areas which had been drawn around single cells as well as clear background. The images were converted to mean intensity values allowing for ratios to be calculated. These ratios were used to determine the change in interaction between POPDC1 and TREK1 in various conditions.

### 4.10 Intact SAN preparation

Experiments were performed on 4-month-old C57Bl6 male mice in accordance with the European Community guiding principles in the care and use of animals (2010/63/UE), the local ethics committee (CREEA Ile-de-France Sud) guidelines and the French decree no. 2013–118 on the protection of animals used for scientific purposes. Mice were anesthetized by intraperitoneal injection of sodium pentobarbital (150 mg/kg). The heart was quickly removed and placed in Tyrode solution containing (in mM): NaCl 140, KCl 5.4, CaCl2 1.8, MgCl2 1, HEPES 5, and glucose 5.5, pH 7.4, titrated with NaOH. The SAN was identified as being bordered by the crista terminalis, the superior and inferior vena cava and the interatrial septum. The SAN surrounded by atrial tissue was dissected and pinned down with the endocardial side up in custom-made optical chambers as described previously [65].

### 4.11 Ca^2+^ Imaging

To measure spontaneous Ca^2+^ transients, the tissue was loaded with 30 μM fluo-4 AM during 1 h at 37 °C, 300 rpm together with 5 μM disrupting or scrambled peptide. 2-D confocal images were recorded in individual SAN cells embedded within the intact SAN at 37 °C, using a resonant scanning confocal microscope Leica SP5, equipped with a white laser fitted to 500 nm. Excitation was collected at >510 nm. Fluorescence traces were expressed as F/F_0_, where F is the fluorescence signal and F_0_ the diastolic fluorescence. Average cycle length was calculated as the time between two consecutive spontaneous Ca^2+^ peaks.

### 4.12 Cell Optiq

#### 4.12.1 Preparation of ARVM for Cell Optiq

Left ventricular cardiomyocytes were isolated from 12-week of New Zealad white rabbits and plated on glass bottom microwell dishes (35 mm petri dish, 14 mm Microwell; MatTek®). Cells were allowed to settle for 2 h prior to each experiment. Cells were treated with 10 μM disruptor peptide or 10 μM scrambled peptide for 2 h at 37 °C prior to measurements being carried out.

#### 4.12.2 Measurements of action potential and contractility

CellOPTIQ® (Clyde Bioscience Ltd.; Glasgow, UK) software was used in the collection of high-speed images of contracting single ARVM. For this set of experiments, contractility and action potential parameters were evaluated. Cells were paced with electrodes at 40 mV with 20 ms duration at a frequency of 1 Hz. For each cell a 30 s recording was taken recording at 100 frames per second using a 60× objective lens.

Trace analysis was carried out using CellOPTIQ software. The unfiltered trace was baseline-subtracted, filtered using 3-point, 15 pass filter. The upstroke and repolarisation phases were filtered adaptively to target smoothness and measured by number of curve inflections. Values used in analysis were Trise (upstroke 10%–90% depolarisation) and action potential duration (APD) 30,50,75,90 values, representing time (ms) from upstroke to various degrees of repolarisation. For example, APD50 represents the time from 50% contraction to 50% relaxation. These were plotted to allow visualisation of the repolarisation phase.

Contraction measurements in cardiomyocytes were achieved using measurements of sarcomere length. High-speed (100 frames per second) and high resolution (2048 × 2048 pixels) video was recorded. Image sampling duration allowed for a minimum of 5 beats to be recorded and subsequently averaged for analysis. Contraction duration (CD50) was recorded, considering both contraction and relaxation. CD50 represents the time from 50% contraction to 50% relaxation of the myocyte. For measurement analysis, the macro programme SarcomereLength created in ImageJ by Dr. Francis Burton (University of Glasgow), was utilized. Each stack of images was analysed by manually positioning a linear selection perpendicular to the cell allowing for the capture of isotropic contractions. The line length was dependent upon cell size and width was kept constant at 50 pixels to reduce any pixel noise.

### 4.13 cAMP phosphodiesterase assay

Recombinant purified MBP-PDE4A4 was incubated with GST-POPDC1 for 30 min prior to the start of this protocol. MBP-PDE4A4 was used consistently at 10 μg for all experiments with the concentration of GST-POPDC1 was increased from 0 to 50 μg. Samples were treated with increasing concentrations of Rolipram from 0.5 μM to 10 μM. The cAMP PDE assay was then carried out as described in [66].

The following are the supplementary data related to this article.

**Figure:**
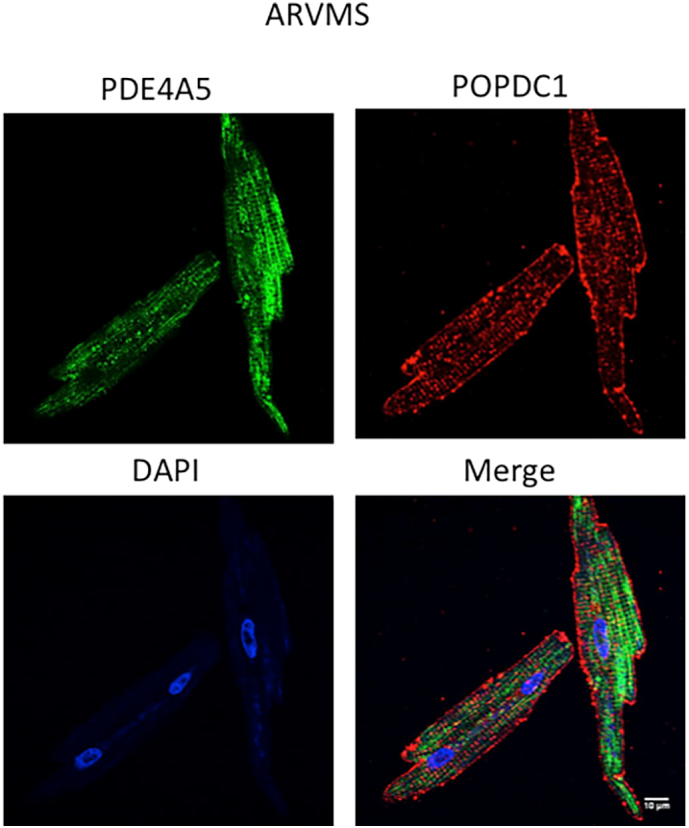
Supplementary Fig. 1. Expression pattern of PDE4A5 and POPDC1 in adult rabbit ventricular myocytes. Immunofluorescence showing colocalization of DAPI (nucleus, blue), PDE4A5 (green: PDE4A5 specific polyclonal) and POPDC1 (red: POPDC1 polyclonal) in ARVMs. (Pearson's coefficient = 0.21).

**Figure:**
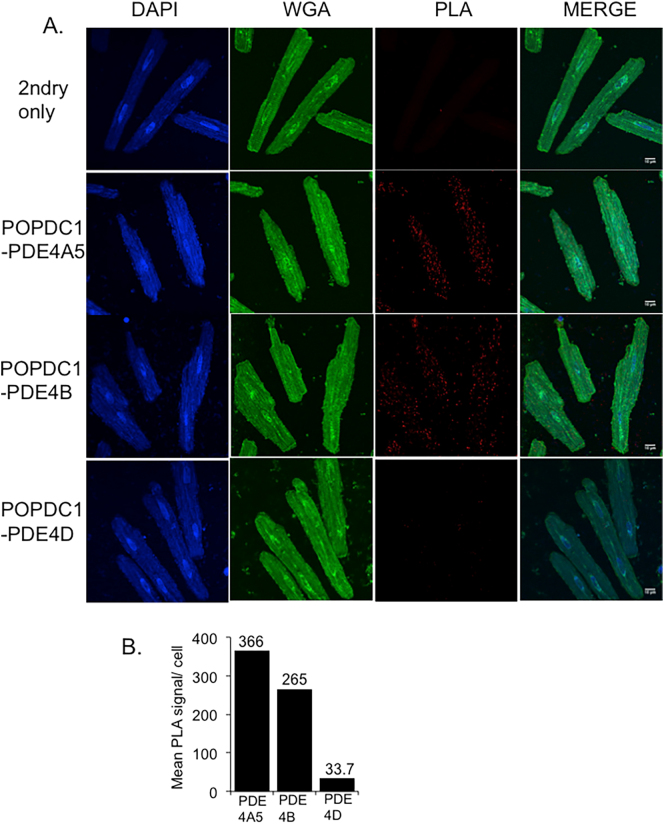
Supplementary Fig. 2. Expression pattern of PDE4A5 and TREK-1 in adult rabbit ventricular myocytes. Immunofluorescence showing colocalization of DAPI (nucleus, blue), TREK-1 (green: TREK1 polyclonal) and POPDC1 (red: POPDC1 polyclonal) in ARVMs. (Pearson's coefficient = 0.54).

**Figure:**
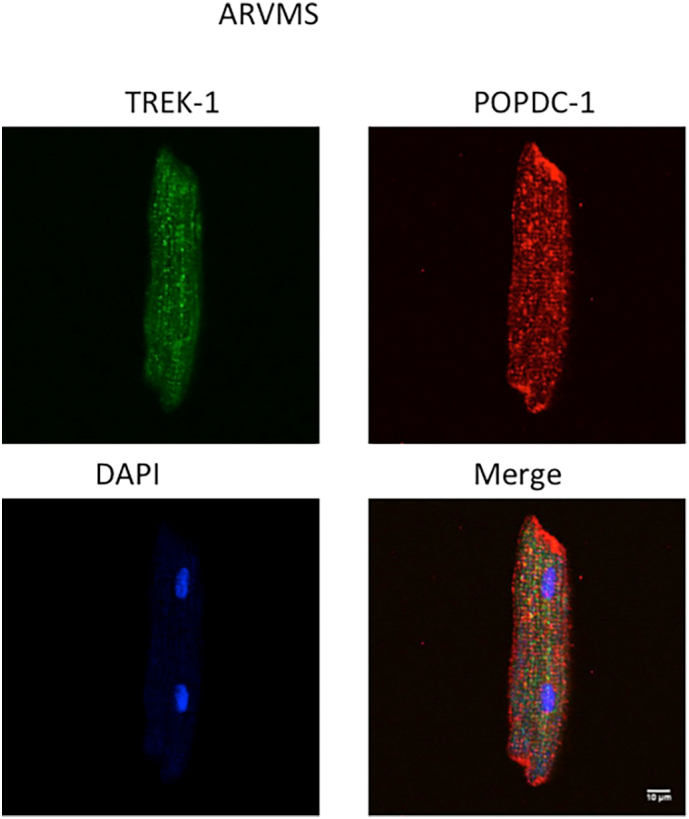
Supplementary Fig. 3. Relative levels of colocalisation between PDE4 and POPDC1 in ARVMS evaluated by PLA. A. ARVM cells were subjected to PLA following staining with antibodies against POPDC1 and either PDE4A5, pan-PDE4B or pan-PDE4D. B. PLA signals from 3 cells were counted for each treatment and a mean value plotted as PLA signals per cell (*n* = 1).

**Figure:**
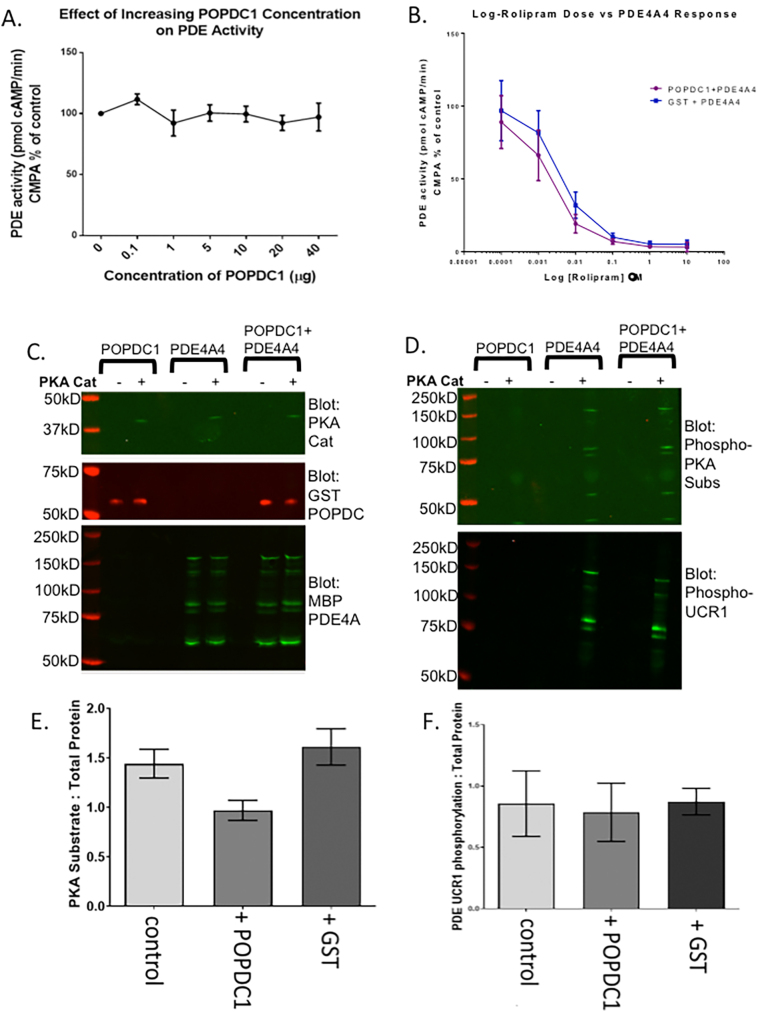
Supplementary Fig. 4. PDE4 activity or phosphorylation by PKA is not affected by POPDC1 association. A. Increasing amounts of Purified recombinant POPDC1-GST and PDE4A4-MBP were incubated together in a PDE assay. The activity of PDE4A4 was recorded. Graphs were constructed using GraphPad Prism 6™. Results are displayed in pmol cAMP/ min and are represented as a mean ± SEM, *n* = 3. B. Purified recombinant POPDC1-GST (purple) or GST (blue) were incubated with PDE4A4-MBP in a PDE assay containing increasing concentrations of Rolipram (blue). The activity of PDE4A4 was recorded and graphs were constructed using GraphPad Prism 6™. Results are represented as a mean ± SEM, n = 3. C. Equal molar concentrations of POPDC1-GST and PDE4A4-MBP were incubated with PKA catalytic subunit. Samples were separated by SDS-PAGE and probed via western-blotting for indicated proteins. D. The gel depicted in C. was blotted for phosphorylated-PKA substrates and a phospho-PDE4 UCR1 to detect changes in the level of PDE4 phosphorylation. E. Intensities from phospho-PKA substrate blots were evaluated using ImageJ and normalised to controls. n of 3, mean ± SEM. F. Intensities from phospho-UCR1 blots were evaluated using ImageJ and normalised to controls. n of 3, mean ± SEM.

**Figure:**
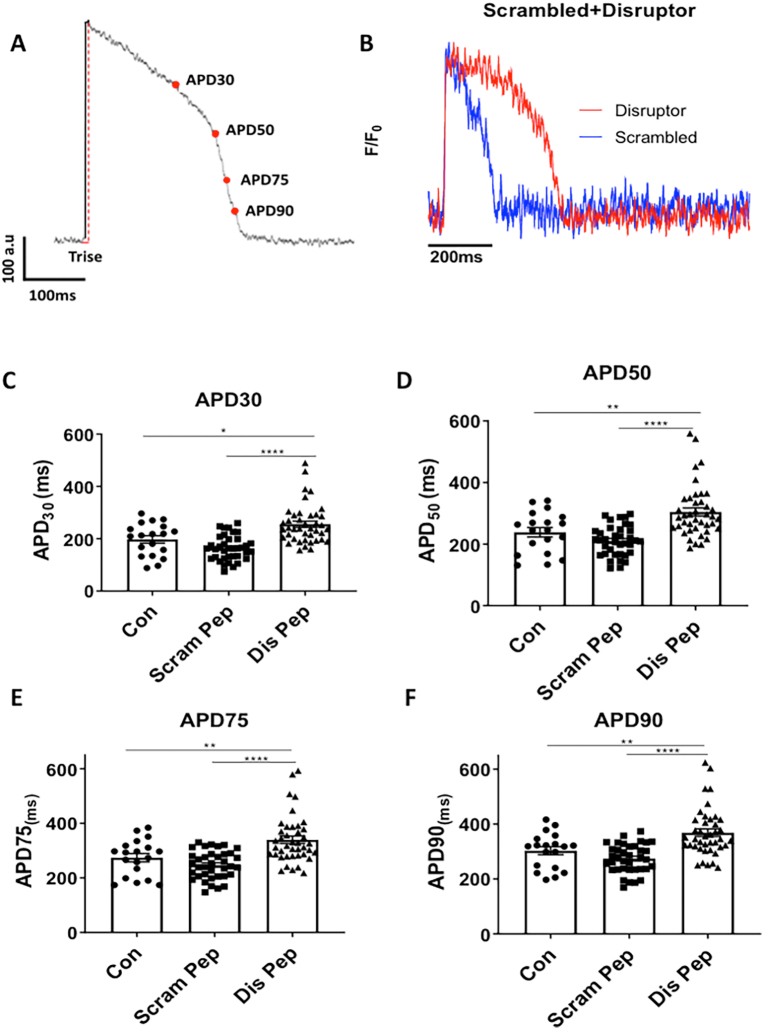
Supplementary Fig. 5. The POPDC1-PDE4 complex influences action potential duration. A. Measurements were collected from 4 points during the repolarization phase. The action potential duration (APD) of ARVMs was determined following treatment with the POPDC1-PDE4A disruptor and scrambled control peptides. B. A representative trace of action potentials with scrambled control peptide and disruptor peptide. Measurements of APD across the action potential repolarization phase (C) APD30, (D) APD 50, (E) APD75, and (F) APD90 were collected from cells which were paced at 1 Hz under baseline conditions and displayed on scatter plot graphs with bars ± SEM. Untreated cells, *n* = 19, scrambled peptide *n* = 37 and disruptor peptide *n* = 42 cells. One-way ANOVA with Tukey's post-hoc analysis, performed using GraphPad Prism™ ****p < 0.0001, **p < 0.01. E. (F) Contraction duration at 50% contraction to 50% relaxation was measured after 2 h of treatment with either 10 μM scrambled or disruptor peptide. Each point represents the mean value from one cell. From one rabbit. One-way ANOVA with Tukey's post-hoc analysis.

**Figure:**
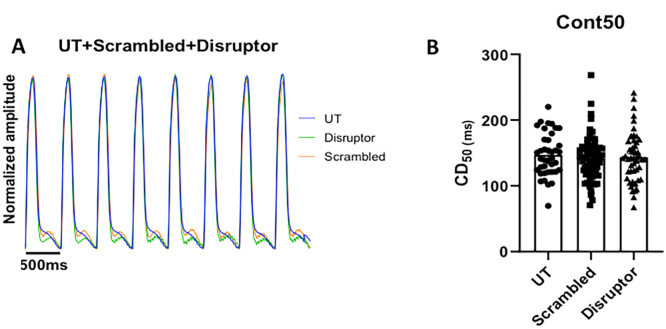
Supplementary Fig. 6. Measurements of contraction in NRVMS. A. Representative traces of contraction. UT = untreated, Scrambled = scrambled peptide control, disruptor = disruptor peptide B. Contraction duration at 50% contraction to 50% relaxation was measured after 2 h of treatment with either 10 μM scrambled or disruptor peptide. Each point represents the mean value from one cell. Untreated cells, *n* = 39, scrambled peptide *n* = 67 and disruptor peptide *n* = 50 cells. From 2 rabbits. One-way ANOVA with Tukey's post-hoc analysis.

**Figure:**
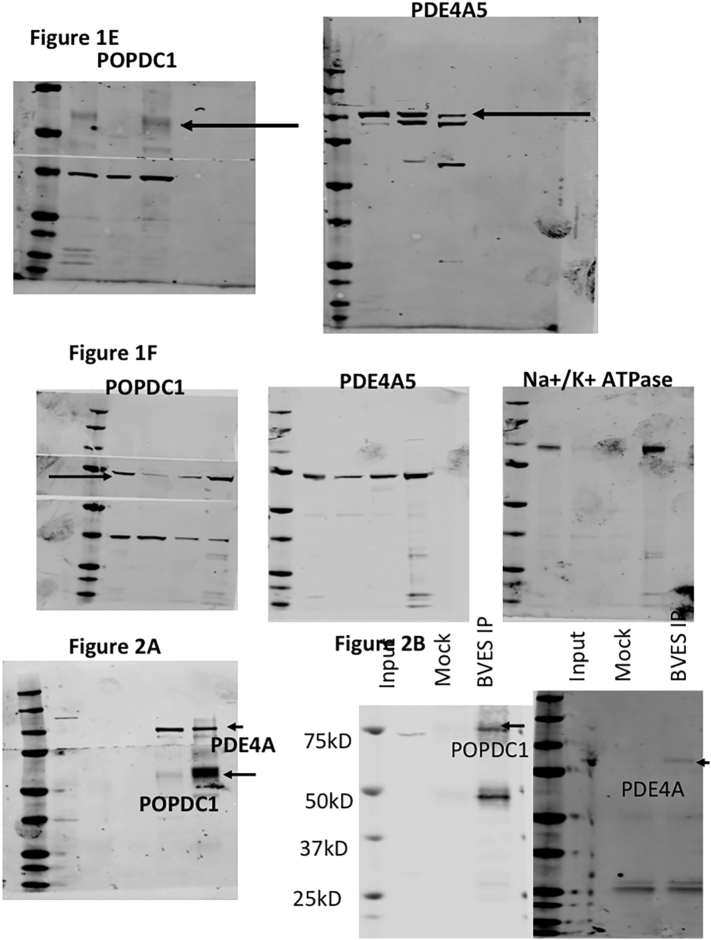
Supplementary Fig. 7. Full gels for Figs. 1E, F, 2A and B. Note that nitrocellulose for Figs. 1E, F and 2A was split for concomitant western blotting of proteins at different weights.

**Figure:**
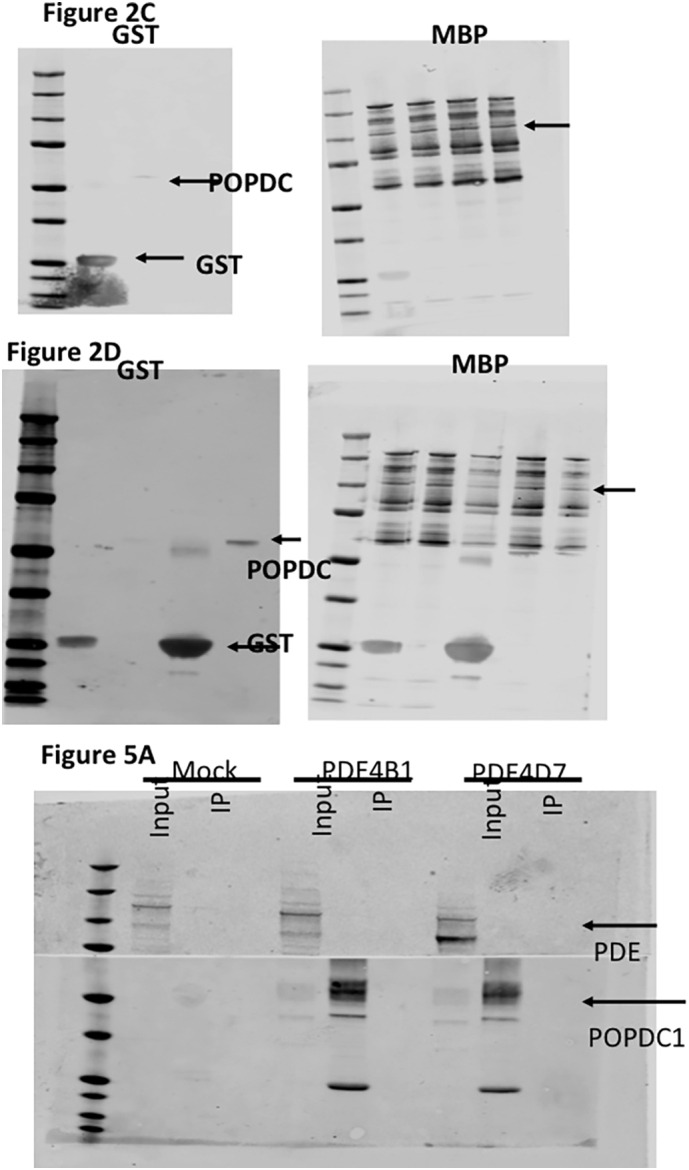
Supplementary Fig. 8. Full gels for Figs. 2C, D and 5A. Note that nitrocellulose for Fig. 5A was split for concomitant western blotting of proteins at different weights.

**Figure:**
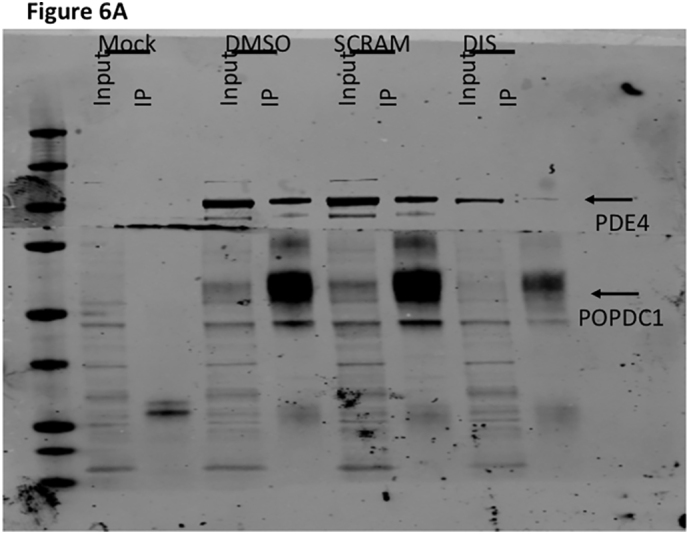
Supplementary Fig. 9. Full gel for Fig. 6A. Note that nitrocellulose was split for concomitant western blotting of proteins at different weights.

## Author contributions

GSB, BS and AJT conceived the project and wrote manuscript with input from TB, WF and GLS. AJT undertook the majority of experimental work with help from AM, JL, CG, CB, GST and RM, CG. GLS, SD and NM planned and carried out all functional myocyte experiments. DM and GV planned and carried out experiments in sino-atrial node tissue.

## Data availability

The data underlying this article will be shared on reasonable request to the corresponding author.

## Declaration of Competing Interest

The authors declare no conflicts of interest.
